# Using epigenomics to understand cellular responses to environmental influences in diseases

**DOI:** 10.1371/journal.pgen.1010567

**Published:** 2023-01-19

**Authors:** Julia J. Wattacheril, Srilakshmi Raj, David A. Knowles, John M. Greally

**Affiliations:** 1 Department of Medicine, Center for Liver Disease and Transplantation, Columbia University Irving Medical Center, New York Presbyterian Hospital, New York, New York, United States of America; 2 Division of Genomics, Department of Genetics, Albert Einstein College of Medicine, Bronx, New York, United States of America; 3 New York Genome Center, New York, New York, United States of America; 4 Department of Computer Science, Columbia University, New York, New York, United States of America; 5 Department of Systems Biology, Columbia University, New York, New York, United States of America; The University of North Carolina at Chapel Hill, UNITED STATES

## Abstract

It is a generally accepted model that environmental influences can exert their effects, at least in part, by changing the molecular regulators of transcription that are described as epigenetic. As there is biochemical evidence that some epigenetic regulators of transcription can maintain their states long term and through cell division, an epigenetic model encompasses the idea of maintenance of the effect of an exposure long after it is no longer present. The evidence supporting this model is mostly from the observation of alterations of molecular regulators of transcription following exposures. With the understanding that the interpretation of these associations is more complex than originally recognised, this model may be oversimplistic; therefore, adopting novel perspectives and experimental approaches when examining how environmental exposures are linked to phenotypes may prove worthwhile. In this review, we have chosen to use the example of nonalcoholic fatty liver disease (NAFLD), a common, complex human disease with strong environmental and genetic influences. We describe how epigenomic approaches combined with emerging functional genetic and single-cell genomic techniques are poised to generate new insights into the pathogenesis of environmentally influenced human disease phenotypes exemplified by NAFLD.

## Introduction

Many human diseases have a clear environmental contribution. For decades, it has been assumed that the environment can influence the regulation of gene expression through “epigenetic” mechanisms, which can be interpreted as meaning the molecular regulators of transcription, such as DNA methylation and chromatin states. The extracellular environmental cue acting to modify epigenetic states has even been given its own name, the “epigenator” [1]. Evidence supporting this model has mostly come from findings of altered epigenetic patterns following environmental exposures. It is a widely accepted model that epigenetic alterations are the primary mediators of environmental influences, potentially propagating these effects long after the cessation of exposure because of the assumed property that epigenetic regulation can self-propagate through cell division.

We have, however, become more critical in our interpretation of genome-wide studies of epigenetic mediators, described as epigenome-wide association studies (EWAS). We have assumed that a change in a transcriptional regulator like DNA methylation, found when comparing pools of cells, reflects individual cells undergoing reprogramming. This interpretation is now recognised to be too simplistic and ignores other ways the same outcome can occur. Evidence linking environmental exposures with epigenetic modifications needs to be reassessed as part of this updated perspective.

In this review, we take a fresh look at the evidence for epigenetic mediation of environmental influences. We use nonalcoholic fatty liver disease (NAFLD) including nonalcoholic steatohepatitis (NASH), as our disease focus. NAFLD is an excellent paradigm for a common disease about which much is known in terms of environmental and genetic risk factors. We describe how NAFLD has been studied to date with exploratory EWAS as an example of how the oversimplified interpretation of results may be misleading.

The constructive way of thinking about the nonepigenetic effects influencing our interpretation of EWAS is that they are not merely impediments to interpretation, but instead offer alternative insights into disease pathophysiology. We will review how a systematic change in either a cell subtype proportion or a genetic variant can lead to a DNA methylation, transcriptional or other changes that we collectively refer to as “molecular genomic” processes, in preference to the ambiguous term “epigenetic.” Such systematic changes reflect cell subtype or DNA variant associations with the exposure or disease and would be valuable to understand as potential contributors to the phenotype. The study of the relationship between genetic and molecular genomic variation is typically described as functional genomics and encompasses the identification of sequence variants that influence molecular genomic processes, including gene expression levels (expression quantitative trait loci (eQTLs)), DNA methylation (meQTLs), and chromatin accessibility (caQTLs). Some functional variants are only revealed following an environmental exposure, uncovering a potential way that individuals can differ in their responses to the same environmental challenges [2]. Furthermore, now that we are in the era of increasingly sophisticated single-cell genomic assays, we are finding unexpected heterogeneity within canonical cell types, and effects of functional sequence variants that are restricted to subtypes of cells [3], representing unprecedented insights into how environmental influences act within a tissue.

It is now timely to rethink how we use molecular genomic assays to understand how the environment influences cells, and how this influence can vary because of differences in the DNA sequence between individuals. NAFLD is a useful focus, representing a disease of major public health importance with strong environmental influences, multiple extrahepatic manifestations, and variable interindividual susceptibility to disease development and progression. For example, the fibrosis that is part of more advanced NAFLD is influenced by glucose as an environmental agent and represents a major target for intervention to prevent the morbidity and mortality of end-stage liver disease. The goal of this review is to prompt innovative ways of thinking about how to gain therapeutic insights into environmentally driven diseases such as NAFLD, using novel molecular genomic and cellular approaches.

## The epigenome and the environment

### Foundational studies

In this review, we define the environment as the influences extrinsic to and influential for the cell, tissue, or organism. Epigenomic studies can be defined as genome-wide mapping of molecular mediators of transcriptional regulation, given that such mediators have been traditionally described to have “epigenetic” properties [4].

An inherent property of epigenetic regulation of transcription is its reversibility—if a gene is activated, it can be subsequently switched off, or vice versa. Molecular regulators of transcription such as chromatin states, DNA modifications, and transcription factor (TF) activity alter as part of cellular differentiation, demonstrating this molecular malleability.

The ability of the same DNA to behave in different ways in different cell types appears to indicate a layer of information residing on top of the DNA sequence, for which the back-translation of epi- (above, upon) -genetics (DNA sequence) seemed a useful descriptive term. It has also become commonplace to use the word “epigenetic” to describe events that appear to occur variably in cells and organisms in ways that are not mediated by DNA sequence variability, making “epigenetic” synonymous with “nongenetic” in a further use of the term.

The idea of a regulatory layer of information, acting to influence cellular properties without causing DNA changes, puts molecular epigenetic processes in the spotlight as potential mediators of differences between cells or organisms that share the same DNA sequence. The puzzle of discordance of monozygotic twins for conditions that clearly had a genetic contribution prompted speculation that the environment could be different (nonshared) for each twin and that this difference in exposures caused only one to develop the disease [5]. Early studies of DNA methylation showed what appeared to be a progressive difference with age between monozygotic twins [6], an association that suggested a mechanism for this discordance.

Earlier work also supported DNA methylation responding to environmental influences. In 1984, rats were fed a methyl-deficient diet with the idea that it would promote neoplasia. The diet was associated with the development of decreased DNA methylation in the livers of the animals [7]. An influential model was the “viable yellow” mouse, animals with a mutation with variable effects on phenotype, ranging from no apparent phenotype at all (pseudoagouti) to a phenotype of yellow fur, obesity, hyperinsulinaemia, and an increased rate of malignancies. In 1998, Wolff showed that feeding pregnant dams a diet designed to increase DNA methylation increased the proportion of pseudoagouti offspring [8], subsequently shown to be associated with increasing DNA methylation at the mutation site, the insertion of an IAP retroelement upstream of the *Nonagouti* gene [9,10]. This was a fascinating paradigm, indicating that maternal diet during pregnancy could influence the eventual adult phenotype of her offspring, and represents part of the foundation for the field of the Developmental Origins of Health and Disease (DOHaD) [11]. The idea that the organism retains a memory of a past exposure involved a different use of the term “epigenetic,” to describe molecular regulatory processes that were heritable from parent to daughter cells [12]. Intrauterine nutritional deprivation during the Dutch Famine of 1944–1945 was described to be associated with obesity in male offspring in young adulthood, limited to those exposed during the first half of pregnancy, whereas exposure in the last trimester and the first months of life was found to have significantly reduced rates of obesity [13]. While the epidemiologists performing these studies were concerned that there could be some confounding effects on these findings, such as the greater fertility and fecundity of women from higher socioeconomic classes [14,15], the model emerged of a foetus adapting to a stressful environment in utero and retaining this memory of exposure in a way that is maladaptive postnatally. This finding became a model for epigenetic mediation of environmental exposures long after the environmental exposure was no longer present.

It would be misleading to give the impression that the only discoveries in this field were being made in mammals or in more economically developed countries. In 1966, Madeleine Charnier, working at the University of Dakar, Senegal, found that sex determination in a reptile species (the rainbow Agama lizard, *Agama agama*) depended on the temperature at which the embryo develops [16]. Temperature-dependent sex determination occurs in many amphibians and fish, another example of a phenotype dependent on environment and not determined by DNA differences. Plant biology was revealing numerous striking examples of exposures apparently being “remembered” by the organism, such as vernalisation, the response of plants to the prolonged cold exposure of winter, which was found to induce the silencing of the *FLC* gene [17], thus maintaining a memory of the past exposure.

The result of these and other observations was a model of environmental influences acting through “epigenetic” regulatory mechanisms, self-propagating their new patterns of organisation to maintain a memory of the past exposure.

## How molecular regulators of transcription may respond to the environment

### DNA and histone modifications

“Epigenetic” molecular regulators of transcription are numerous. The pattern throughout most of the mammalian genome is one of DNA wrapped around octamers of histones to form a nucleic acid:protein complex called a nucleosome. Most of the cytosines located at CG dinucleotides genome-wide are modified by the addition of a methyl group to form 5-methylcytosine. Where the genome departs from these default patterns typically represents the locations of regulatory elements, where sequence-specific proteins like TFs bind to the DNA. The histones in the flanking nucleosomes acquire patterns of posttranslational modifications (PTMs) that flag these sites as promoters, enhancers, or other regulatory elements. Further distinctive patterns of DNA and histone modifications are found at loci undergoing transcription, while repressive histone modifications or histone variants represent a more macro level of organisation, defining heterochromatic regions of the genome.

DNA methylation remains the paradigm for a transcriptional regulator that can maintain a biochemical memory through cell division, as 5-methylcytosine can be propagated from a parent chromatid to both daughter chromatids. There is also evidence for propagation of a repressive histone modification (histone H3 lysine 27 trimethylation, H3K27me3) through a number of generations of the nematode *C*. *elegans* [18]. TFs appear to remain bound to their DNA target sites through DNA replication, a phenomenon described as “mitotic bookmarking” [19], representing a further way that memory of the molecular organisation of the chromatid in a parent cell can be passed to replicated chromatids in daughter cells.

We assume that a cell is faithfully able to propagate its transcriptional regulatory organisation to daughter cells as a way of transmitting the memory of a prior environmental exposure. However, there is another factor worth considering—it appears that some histone modifications and histone variants can directly mediate responses to the environment. In **Table 1,** we summarise some of these possible environmental influences and molecular regulatory responses.

**Table 1:** Examples of environmental exposures that directly affect molecular regulators of transcription.

Environmental exposure	Molecular regulatory changes	Effect	Genomic location of effects	Reference
Glucose (through hexosamine biosynthetic pathway)	GlcNAcylation of histones	Unclear	Unclear	(See footnote*)
	GlcNAcylation of EZH2	Enhances protein stability and enzymatic activity	Heterochromatin (H3K27me3)	[20]
	GlcNAcylation of TET enzymes	Enhances TET1 activity	Loci with 5-hydroxymethylcytosine	[21]
Lactic acid (anaerobic metabolism)	Lactylation of histones	Possibly gene activation	Active gene promoters	[22]
Serotonin	Serotonylation of histones	Possibly enhanced gene expression	Gene promoters	[23]
Ethanol	Acetylation of histones	Induction of expression of genes involved in signal transduction and learning and memory	New loci of H3K9ac and H3K27ac formed (promoters/enhancers)	[24]
NAD+ metabolism	Enhanced SIRT1 activity	Protein (histone) deacetylation	Unclear	(See footnote**)
	MacroH2A	Reverse effect: macroH2A1 binds to PARP-1 and limits its availability in the cell	None expected	[25]
Hypoxia	G9A	Increased protein stability	Gene promoters	[26]
	KDM5A	Inhibition of activity	Loci with H3K4me3 and H3K36me3	[27]
Short chain fatty acids (e.g., produced by gut fermentation of dietary fibre)	Inhibition of histone deacetylases (HDACs)	Increased histone crotonylation	Histone crotonylation is enriched at gene promoters	[28]
Dietary folic acid, vitamins B_6_ and B_12_	Increased production of S-adenosyl methionine (SAM)	Increased methylation of DNA and histones	Genome-wide	[29]
Vitamin C	TET cofactor	Increased 5-hydroxymethylcytosine, decreased 5-methylcytosine	Genome-wide	[30]

*While GlcNAcylation of histones is often described as an example of a metabolic sensor in chromatin, the concern has been raised that GlcNAcylation does not occur on mammalian histones [31]. More information appears to be needed to resolve this issue.

**The relationship between NAD+ and SIRT1 activity has been debated for a long time, as described in this review [32].

### Two molecular genomic problems to resolve: Cellular memory and sequence specificity

These observations indicate that the environment may be able to act through regulators of chromatin and modifiers of DNA to influence gene expression. While these are intriguing findings and provide clear candidates for the mediation of environmental effects on transcriptional regulation, there are two problems that remain to be overcome before invoking any as primary mediators of long-term, stable cellular reprogramming.

The first is cellular memory. With the exception of DNA methylation, there are no clear biochemical mechanisms for self-propagation of the specific molecular events in **Table 1** to daughter chromatids following cell division. What needs to be invoked to support the idea that these chromatin constituents have long-term consequences is a two-step model, one involving an initial environmental perturbation, followed by the maintenance of a new equilibrium of transcriptional regulators that have the capacity to maintain their patterns long term and through cell division. For example, is it possible that a hyperglycaemic event that increases O-GlcNAcylation acutely then induces EZH2 activity nearby in the genome, causing de novo patterns of formation of H3K27me3 that are then maintained, even after the hyperglycaemia subsides?

The second problem is that these global regulators of transcription lack sequence specificity and therefore do not have the ability to choose specific loci for selective activity. Once again, a separate mediators has to be active initially, for example, when short chain fatty acid exposure causes specific subgroups of promoters to be selected for increased histone crotonylation [28]. Later, we make the case that this is likely to be mediated by sequence-specific TFs establishing regulatory patterns in response to environmental challenges, patterns that are passed on to the global regulators of transcription, allowing TFs to have a primary role in the model of epigenomic responsiveness to environmental stimuli.

## Epigenetic association studies

### Epigenetic studies and NAFLD

When attempting to understand whether epigenetic changes occur associated with traits or disease phenotypes, an EWAS has been the typical approach. As introduced in **Box 1**, an EWAS is generally performed on multiple individuals in a test group (with the phenotype) and a control group that is matched for potentially influential variables like age, sex, and ancestry. The same tissue type is sampled in all individuals, and an epigenome-wide assay is performed, usually studying DNA methylation at numerous loci in the genome, ranging from tens of thousands to millions of sites. For environmental studies, testing the same individuals in conditions when exposed and not exposed is an alternative study design that eliminates the influence of genetic variability, as discussed later, and it may be possible to quantify the exposure so that you do not have to compare two groups, but can instead correlate exposure as a continuous variable with the DNA methylation changes. When there is a locus or set of loci that has a difference of DNA methylation that appears statistically nonrandom, this represents a positive outcome for the EWAS. Generally, the result of a positive EWAS is one of numerous loci showing significant differences in DNA methylation, which generally leads to an attempt to interpret why this group of genomic regions underwent regulatory changes, involving linking each site with a gene, and then looking for a coherence of properties of these genes, using gene ontology or pathway information.

**Box 1. The epigenome-wide association study (EWAS).**

The epigenome-wide association study (EWAS) refers to the testing of samples from individuals with a phenotype or exposure of interest using an assay testing the molecular regulators of gene transcription. This broad picture narrows significantly in practice, as most EWAS involve testing human blood leukocytes and the use of DNA methylation microarrays to survey across the entire genome. “Epigenome-wide” should not be taken to indicate comprehensive coverage of the genome, as microarrays typically represent no more than a few percent of the CG dinucleotides at which DNA methylation occurs. The groups are compared for consistent differences in DNA methylation levels between them, a positive outcome reflected by loci showing changes that are statistically significant. The underlying hypothesis driving these studies is the assumption that the phenotype being studied involves a reprogramming of the transcriptional regulation of the cell, not DNA sequence changes. By identifying DNA methylation differences between the groups, we not only gain evidence for this model, we also find the genes involved in changing the properties of the cell as part of the development of the phenotype. As will be discussed in the main text, it is not straightforward to interpret the results of these studies, for a number of reasons. The positive view is that the factors that make EWAS difficult to interpret, if understood, allow insights into the development of the phenotype, but at the expense of the hypothesis of cellular reprogramming.

We are using NAFLD as the focal point for the discussions to follow. NAFLD represents a major complication of the obesity epidemic, with NAFLD now the most common form of liver disease worldwide [33,34], estimated to affect 6 to 30 million people in the United States, including 600,000 who have advanced to developing cirrhosis [35]. A spectrum of histological stages characterizes the disease, ranging from simple fat accumulation (or steatosis) to an inflammatory phenotype, NASH, which can progress to involve fibrosis and cirrhosis [36]. Clinical outcomes with cirrhosis include decompensation, portal hypertension, liver transplantation, hepatocellular carcinoma, and death. Long-term follow-up of NAFLD patients confirms that NASH patients have a higher risk of liver-related mortality than non-NASH patients [37]. The economic burden of NASH is significant—the lifetime costs in the USA for NASH patients alone in 2017 was estimated to exceed US$200 billion [38].

The pathogenesis of NAFLD involves both environmental exposures and genetic predisposition [39,40]. The causative environmental exposures are generally involved in causing obesity, such as the Western diet, but distinct gut microbiome complements in NAFLD patients are also invoked as possible contributory exposures [41]. It comes as no surprise that DOHaD models that are linked to obesity are also linked to NAFLD, with exposures during foetal and early neonatal life to maternal under- and overnutrition, excess glucocorticoids, and environmental pollutants linked to offspring NAFLD [42]. Whether these exposures act primarily to cause offspring obesity, with NAFLD as a secondary consequence, or whether early developmental programming predisposes to liver damage independently remains unclear.

Genetic susceptibility is a factor of importance in the development of NAFLD, which has a strong heritable component and involves several known loci [43]. NASH can develop in lean individuals, especially when carrying genetic risk alleles [44]. DNA sequence changes identified through genome-wide association studies (GWAS) have been causally associated with NAFLD [45–48], discussed in more detail below.

**Fig 1:**
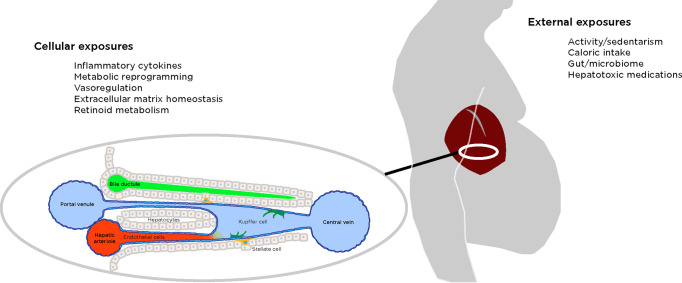
Environmental exposures associated with disease are not necessarily the same when considering those extrinsic to the organism, and those directly acting on the cellular microenvironment, depicted with examples relevant to NAFLD.

NAFLD helps to illustrate an important point—the environmental exposure extrinsic to the organism may not be the same exposure experienced by the cells in the affected tissue (**Fig 1**). In the case of NAFLD, the exposure extrinsic to the cell are the lipotoxic lipid species that influence hepatocytes in a poorly understood stress model. More relevant is the extrinsic exposure that causes NAFLD to progress to significant fibrosis, exemplified by high-fructose corn syrup [49,50], while the equivalent cellular exposure inducing fibrosis is the inflammatory cytokine TGFß [51].

We describe below several studies demonstrating tissue- and genomic region-dependent variation in DNA methylation in NAFLD, the foundation for proposals that DNA methylation has a role in the pathogenesis of NAFLD [52]. There have been several human EWAS performed on cohorts of patients with NAFLD that are worth examining as examples of a broader field of use of epigenomic assays to study environmental effects. Studies involving DNA methylation assays were chosen as this is at present by far the most commonly used epigenomic assay in EWAS. In **Table 2,** we list eight studies involving DNA methylation studies of hepatic steatosis. None of these include extrahepatic phenotyping and are instead focused solely on liver disease.

**Table 2:** Examples of prior EWAS performed in NAFLD and NASH.

Phenotype(s)	Number of individuals studied	Tissue type(s)	Epigenomic assay(s)	Citation
NAFLD: mild	33	Liver	DNA methylation	[53]
NAFLD: severe	23		Gene expression	
Normal controls	18	Liver	DNA methylation	[54]
Healthy obese	18		Gene expression	
NAFLD	12			
NASH	15			
Serum GGT, AST, and ALT levels	731 (discovery)	Peripheral	DNA methylation	[55]
Hepatic steatosis (ultrasound of replication cohort)	719 (replication)	blood		
Healthy obese	35	Liver	DNA methylation	[56]
NAFLD	34		Gene expression	
NASH	26			
Normal controls	30	Peripheral	DNA methylation	[57]
NAFLD	35	blood		
NAFLD: mild	35	Liver	DNA methylation	[58]
NAFLD: advanced	25			
Normal controls	15	Liver	DNA methylation	[59]
NAFLD and fibrosis	15		Gene expression	
Healthy controls	30	Peripheral	DNA methylation	[60]
NAFLD	18	blood		
NASH	17			
NAFLD: severe fibrosis	119	Liver	DNA methylation	[61]
NAFLD: no fibrosis	206			

EWAS, epigenome-wide association studies; NAFLD, nonalcoholic fatty liver disease; NASH, nonalcoholic steatohepatitis.

### EWAS design issues

These studies can be used to help illustrate some of the strengths and weaknesses of epigenetic association studies, as have been extensively reviewed in prior publications [62–65]. We break down some of the major issues in the following five categories.

#### Surrogate tissue sampling

Some human tissues are very accessible (e.g., peripheral blood leukocytes, buccal epithelium, hair follicles), but many common diseases are mediated by organs that would require sampling using invasive and risky procedures (e.g., liver, lungs, brain). A frequent question is whether an accessible tissue like peripheral blood leukocytes can report the epigenetic events occurring in an inaccessible tissue in the same individual [66,67]. What tends to be missing in EWAS projects using surrogate tissues is a clear rationale at the outset for the choice of the tissue used. If all that is required from the study is the development of a robust biomarker, in which DNA methylation changes reflect or predict a disease but without the need to understand why the DNA methylation change occurred, the use of surrogate, easily accessible tissues is highly desirable. If the question is whether the mechanism of the disease can be revealed through studies of a tissue like peripheral blood, that is when the rationale should be explicit—is the assumption that all cells in the body are changing their transcriptional regulatory patterns in the same way, both in the accessible and inaccessible tissues? Three of the NAFLD studies in **Table 2** use peripheral blood leukocyte DNA for DNA methylation assays [55,57,60], making these studies examples of the use of surrogate reporter tissues. For their results in peripheral blood leukocytes to be informative about the physiology of cells in the liver, the genes and pathways would have to be active in both of these very distinctive tissue types and dysregulated in the same manner in response to an environmental exposure that may or may not be comparable in these distinct tissues. At present, such an explicit description of the rationale for the study or any discussion about whether this model is realistic is typically absent from reports of epigenetic studies of surrogate tissues.

#### Cohort sizes

The median number of individuals in each cohort in **Table 2** is 65, falling to 61.5 in the studies using liver samples. Obtaining well-characterised, high-quality liver samples for molecular studies is very challenging, reflected by these limited numbers. The question about how many samples are needed to power an epigenetic association study is frequently raised [68,69]. There are three major interacting variables that can be considered—the degree of change of DNA methylation, the number of sites tested, and the number of samples compared. When you use fewer samples, you can only confidently attribute changes of DNA methylation that are of greater magnitude and at fewer sites. It is a reasonable conclusion that these studies in **Table 2** detected only a limited subset of the total likely number of DNA methylation changes occurring in these individuals.

#### Reverse causation

A further issue is whether the DNA methylation changes can be assumed to cause the phenotype, or whether they are caused by the phenotype, the latter being an example of “reverse causation.” This is a limitation inherent to cross-sectional study designs in which one group compared already has the phenotype [64]. Examples of reverse causation include obesity-related phenotypes that have been found to change DNA methylation of peripheral blood leukocytes (studying body mass index (BMI) [70,71] or blood lipid profiles [72]). Reverse causation represents another way that we can overinterpret epigenetic association studies, by making the incorrect assumption that the DNA methylation changes are causing the phenotype.

It has been proposed that a good way of addressing this issue of confounded interpretation of reverse causation is through the use of a more difficult study design: longitudinal sampling of the same patients over time [73]. The Ahrens study in **Table 2** accomplished this in a clever manner, sampling liver before and after bariatric surgery [53]. They found DNA methylation changes to be partially reversible at a subset of loci when liver biopsies were compared after bariatric surgery and associated dramatic weight loss (averaging 40 kg per person). By including a “healthy obese” group in their study, the possibility that the environmental exposure of obesity represents an independent and confounding influence on DNA methylation in the liver is addressed, allowing a focus on a subset of loci that is more likely to be involved in disease pathogenesis [54].

#### Cell subtype proportional composition

It is possible for cell subtype proportions to vary within the tissue studied and between the groups tested and lead to a result showing changes of DNA methylation without any cells in the samples having undergone molecular reprogramming. For a DNA methylation change to be attributable to this influence, the cell subtype proportional change has to be consistently differing between the groups—in other words, the different proportion of a specific cell subtype needs to be nonrandomly present across the individuals in the test group compared with the controls.

Of the studies in **Table 2**, the Nano group [55] applied the *minfi* software package [74] to account for six leukocyte subtypes in analysing their results of peripheral blood studies, a mainstream approach in EWAS at present. Had cell subtypes been studies in liver itself, multiple changes would be expected, confirmed by the Johnson study that used the *EpiDISH* cell subtype deconvolution approach [75], revealing a decrease in epithelial cells and increases in immune cells, in particular natural killer (NK) T lymphocytes, with the progression of fibrosis in their liver samples. In the earlier stages of NAFLD, reprogramming of cellular properties should be reflected by the accumulation of large amounts of lipids in the cytoplasm of hepatocytes, manifested histologically by the displacement of the nucleus to a peripheral intracellular location. Cell subtype compositional changes would be likely to include the influx of inflammatory cells into the liver parenchyma with disease progression, and the transdifferentiation of hepatic stellate cells to the myofibroblasts that produce the extracellular matrix proteins that cause fibrotic scarring of the organ. The cells composing the liver affected by NAFLD or NASH are therefore substantially different to those in normal, healthy livers, both in terms of their innate properties and the representations of different subtypes. The development of single-cell transcription profiles from these kinds of tissues is likely to generate new ways of testing for both reprogramming and cell subtype proportional changes in disease like NAFLD [76].

#### Genetic effects on DNA methylation

**Fig 2:**
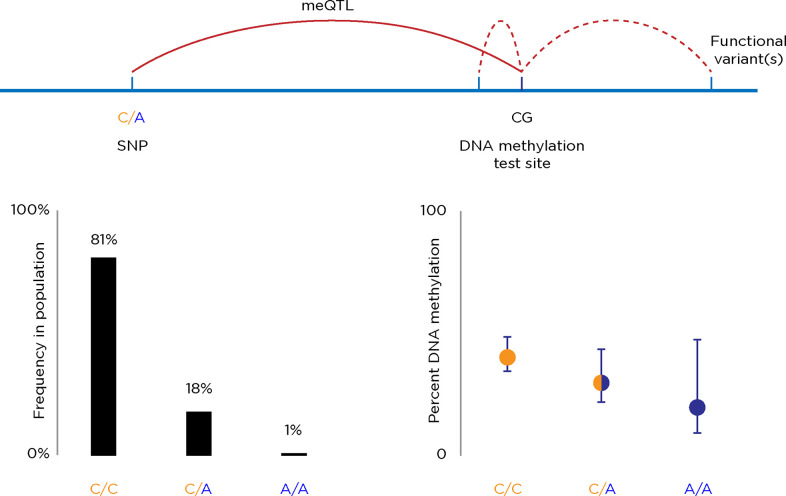
An illustration of a methylation quantitative trait locus (meQTL). These are identified by finding a difference in DNA methylation between individuals that correlates with having one or other allele for a DNA sequence variant, in this case showing a variant on the same chromosome (a *cis*-meQTL). A significant change in DNA methylation associated with these differing allelic states defines the meQTL. Whether the meQTL causes the DNA methylation change is less certain—within the haplotype containing the meQTL and the locus where DNA methylation was tested will include other DNA sequence variants, one or more of which could be directly influencing the DNA methylation as a functional variant.

Finally, the influence of genetic sequence variation between the individuals studied needs to be considered. It is now widely recognised that DNA sequence variability can be associated with DNA methylation differences between individuals, defining functional variants referred to as meQTLs [77]. These are usually revealed when both genotyping and DNA methylation studies are performed on cohorts of individuals, testing whether differences in DNA methylation (the quantitative trait) are associated with the presence of different alleles for a variant at a locus within the flanking 1 Mb. In **Fig 2,** we illustrate the example of a locus with a C/C genotype on both alleles in 81% of people in the population, a heterozygous C/A in 18% and a homozygous A/A in 1%. DNA methylation at a nearby site averages 40% in the C/C individuals, 30% in the C/A, and 20% in the A/A people, revealing a trend in DNA methylation associated with genotype. It may not be the variant itself with the C:A polymorphism that mediates the effect on the DNA methylation, as that locus is transmitted with a substantial amount of flanking DNA, representing a haplotype in which a separate functional variant could also be carried.

When the proportion of DNA methylation variation attributable to DNA sequence variation has been estimated, using different assays, tissue types, and ways of making these estimates, very substantial effects have been found, estimated to vary between 14% and 80% [78–85]. Of the studies in **Table 2**, that by Nano and colleagues was distinctive for identifying which DNA methylation changes were attributable to DNA sequence variation. They found four sites of distinctive DNA methylation that survived their other rigorous filtering criteria, of which three were attributable to DNA sequence variation, leaving them with a single locus near the *SLC7A11* gene where DNA methylation appeared to be associated with hepatic steatosis on its own and not genetic variability between the groups tested [55].

In performing these kinds of EWAS, the ancestries of the patients studied should be described. Two of the **Table 2** studies were on Han Chinese [57,60]; one was on “Caucasian women” [59], and one on Japanese patients [58]. The effect of DNA sequence variability on DNA methylation variability is so strong that ancestry has been shown to be predictable from DNA methylation assays [86]. Furthermore, with clear differences in susceptibility to NAFLD and NASH as complications of obesity between Hispanic and Black American patients [87,88], not accounting for ancestry in study design could be a strong influence on the results obtained if the affected individuals are disproportionately of one ancestral category. The meQTLs in a specific tissue type can differ between individuals of different genetic ancestries [89,90], which could cause the appearance of DNA methylation differences associated with a disease if the individuals studied in the disease group are disproportionately from distinct ancestral origins to the controls. The variability in DNA methylation and gene expression can even occur at fine-scale geographic levels, as demonstrated in a study identifying within-island DNA methylation and gene expression differences in individuals of diverse ancestry in Indonesia [91].

Finally, as mentioned above, if we know something about the genetic susceptibility affecting individuals within the cohort affected by the disease, that information is probably worth including. NAFLD has been found to be associated with protein-coding sequence variation, including the I148M missense variant in *PNPLA3* that compromises the individual’s ability to hydrolyse triglycerides [92]. It would be unwise to assume that this subgroup of patients with NAFLD or NASH is experiencing the same environmental exposures at the tissue and cellular level as individuals with different or no recognisable pathogenic variants. Including protein-coding pathogenic variant information as a likely source of variability in EWAS is prudent as a major influence on phenotypic outcomes. It is likely that the pathogenic burden in the genome (genetic variation contributing to disease) is concentrated in loci of open chromatin in disease-relevant tissues [93]. In this “omnigenic model” of complex traits, genes with modest effects throughout the genome are influencing disease etiology through cell- and tissue-specific effects. This model supports the findings with NAFLD that genetic and epigenetic mechanisms interact with environment in a variable, tissue- and cell-dependent manner that introduces variability to EWAS outcomes.

The nine studies of **Table 2** are used to represent the broader field of EWAS. Like most EWAS, each study has its own strengths, as defined above, but equally no individual study can be said to have addressed all of the problems inherent to how EWAS are currently performed. The potential outcome that results from failing to account for these problems is that changes in DNA methylation could be overinterpreted, assumed to represent cellular reprogramming responding to an environmental provocation, and causative of the phenotype, whereas the DNA methylation change could instead be due to changes in cell subtype composition within the tissue, genetic differences between individuals, or the consequence of the hepatic phenotype.

### The long road from EWAS to individual disease prediction

Both GWAS and EWAS have been touted as having great promise for precision medicine, particularly for polygenic disorders such as NAFLD and NASH [94]. For polygenic traits, risk prediction through a risk score offers one such avenue. Typically, genetic risk scores (GRS; also described as polygenic risk scores (PRS)) are constructed to identify high-risk individuals based on GWAS results. The GRS is calculated as the weighted sum of the risk alleles for a trait in an individual, using weights determined by the best statistically powered GWA study for the trait. GRS have shown mixed utility in the ability to identify high-risk groups of individuals [95], and limited translation across ethnicities [96]. Recent NAFLD GWAS from the UK Biobank have identified >90 associated genomic loci, allowing the development of a GRS that successfully identified high-risk cases as having an odds ratio of 2.1 compared to individuals with the lowest NAFLD GRS score [94]. This difference is more modest than GRS for other diseases, such as the 3-fold higher risk for coronary artery disease [97].

DNA methylation risk scores (MRS) are proposed to capture the effects of the environment in generating a risk score for a phenotype [98]. Like a GRS, the MRS summarises information across multiple informative loci in the genome to generate a single score, but based on risks associated with DNA methylation values, not with genetic variants. There are numerous factors that limit the utility and widespread calculation of MRS. The first is that there are too few large-scale EWAS with concurrent genotyping to provide adequate external datasets to calculate MRS [98]. The DNA methylation arrays may also introduce bias that skews interpretability due to the microarray design. For traits such as NAFLD that involve strong environmental effects, the hope would be that MRS would capture these influences and would offer predictive power beyond sequence variation alone.

It is encouraging that the few genome-wide DNA methylation studies published in recent years suggest that blood DNA methylation measurements provide a stable and accurate assessment of risk when calculating MRS, for a range of phenotypes [99–102]. One recent study including the largest reported MRS cohort (*n* = 831) indicated that DNA methylation scores outperform baseline risk and GRS, improving imputation of 139 outcomes, compared with just 22 improved through GRS [103]. Many of these MRS replicated in external cohorts of different ethnicities and showed variable but robust replication across kidney-related traits in diverse populations. This remains an emerging area of research but looks to have room to improve when the accuracy of MRS is enhanced with increased sample sizes.

## Embracing the sources of variation

### Spurious associations reveal systematic changes

If it sounds daunting to consider the possibility that we need to go beyond the DNA methylation change and understand its cause in terms of cell subtype or DNA sequence variants, there is an important point to consider. For one of these influences to influence the results, sending the DNA methylation in a specific direction so that it differs between groups, the spurious influence needs to be nonrandomly distributed between the groups. For example, if you are testing liver samples and there are more Kupffer cells in samples from one group compared with the other, this will cause the appearance of a DNA methylation change related to the loci that are distinctively methylated in that cell type. Likewise, in the situation of the meQTL above, where the C allele is associated with increased and the A allele with decreased DNA methylation, you will only see a resulting difference in DNA methylation between groups if the C allele is overrepresented in one group and not the other. These typically unrecognized influences, if studied, reveal cellular changes and genetic associations with the disease being studied, which represent potentially valuable insights into its pathogenesis.

### Examples of cell subtype changes following environmental exposures

It should be no surprise that environmental cues prompt responses that involve the composition of a tissue changing in terms of cell subtypes. Endocrine disrupting chemicals are characterised by altering sexual differentiation during development, a stark example where entire organs form differently in response to an environmental exposure.

We have previously highlighted [65] an interesting model of a cell subtype response to vitamin A deficiency during intrauterine development. In this mouse model, the associated tissue phenotype was quite subtle, involving hyperplasia of smooth muscle surrounding distal airways, established during lung development and persisting into adulthood, causing bronchoconstriction in response to a methylcholine challenge [104]. A very similar phenotype that recapitulates a component of the human asthma phenotype was found following prenatal deficiency of vitamin D [105]. While relatively subtle from a histological point of view, the change in cell subtype composition and organisation in the lung tissue was enough to cause a measurable phenotype.

EWAS of environmental exposures have also revealed cell subtype changes in peripheral blood. In a study of low-level intrauterine exposure to arsenic, DNA methylation of umbilical cord blood leukocytes was tested and analysed using an approach comparable to that described to be used by Nano group [55] earlier. These researchers found that arsenic exposure was associated with a higher proportion of CD8+ T lymphocytes [106]. The authors noted that the deconvolution approach based on DNA methylation profiles of adult reference cell types does not work as expected in cord blood, as was later systematically reviewed [107], and could therefore call their results into question. However, a separate study of arsenic exposure to adult mice showed an increase in CD8+ T cell proportions in bronchoalveolar lavage samples [108], suggesting that the exposure has consistent effects to increase this proportion of lymphocytes in different tissues.

A consistently robust association from blood leukocyte DNA methylation studies is with a history of cigarette smoking [109]. One of the loci at which DNA methylation is distinctive in smokers is at the gene for the GPR15 surface marker. When this specific marker was studied further, a complex picture emerged. Initially, it appeared that GPR15 defined a specific T lymphocyte subtype in blood that increased in proportion following smoking [110]. However, the same researchers followed up with a study that showed GPR15 to be present on many T lymphocyte subtypes following smoking, indicating that the protein is induced by cigarette smoke exposure in many cell subtypes and does not necessarily reflect a change in cell subtype proportions, as they had originally proposed [111]. It should be noted that DNA methylation studies of peripheral blood generate a robust biomarker of smoking, a very useful indicator of a relevant environmental exposure when studying a phenotype like lung cancer [112].

When performing studies on human liver tissue, given the increasing availability of single-cell RNA-seq data from both healthy [116] and diseased [117] livers, it should be possible to go back to the results of those studies from **Table 2** that included gene expression analyses of liver tissue [53,54,56,59] and get an indication of what proportion of the observed DNA methylation differences are due to cell subtype changes (**Box 2**). Rather than regarding this as undermining the results of an EWAS, such an additional layer of information potentially enhances insights into disease pathogenesis, by revealing cell subtype proportion changes occurring as part of the development or progression of the disease. Furthermore, if this kind of approach permits removal of DNA methylation changes that are due to cell subtype changes, the remaining signal is much more likely to represent the kind of cellular reprogramming events typically sought in an EWAS.

**Box 2. How can cell subtype proportions be measured?**

The complete blood count and differential white cell measurement provides quantification of lymphocytes, granulocytes (neutrophils, eosinophils, and basophils), and monocytes. This represents a restricted group of major subtypes of white blood cells, recognising that there are many subtypes of lymphocytes in particular, and monocytes are also inherently heterogeneous [113]. If DNA methylation microarray data are available from a tissue and if subtypes of cells have been tested to identify loci within the microarray with distinctive DNA methylation patterns in the different cell subtypes, the data can be analysed in a way that allows estimation of the proportions of each cell type present. This technique has been developed primarily for white blood cells, testing a different set of cell subtypes than is reported by the clinical differential white cell count: granulocytes, monocytes, and four subtypes of lymphocytes, B cells, natural killer cells, and CD4 and CD8 T cells [114]. If gene expression data are available, a comparable approach to allow estimation of cell subtypes can be performed, a salient example being CIBERSORT, which allows 22 different white blood cell subtype proportions to be estimated [115]. When reference gene expression data have not been generated from isolated cell subtypes in a tissue, the results of single-cell transcription studies can instead be used [76], allowing cell subtype estimations to be broadened beyond blood to other organs and tissues.

If the vitamin A or vitamin D deficiency studies of the mouse lung described above were performed by a currently typical EWAS approach, sampling the bulk lung tissue from animals in each exposure group, testing DNA methylation patterns, and removing those changes attributable to cell subtype changes, we would be eliminating from further consideration the smooth muscle changes that mediate the bronchoconstriction phenotype in these animals. Likewise, the finding from DNA methylation studies of an increased proportion of CD8+ T lymphocytes following arsenic exposure is consistent with what has been found through immunological studies of the effects of toxicity of this heavy metal [118]. These cell subtype proportion changes occurring nonrandomly in patients with a disease are not, therefore, an artefact to discard, but instead represent an insight into disease pathogenesis, and should be harvested as useful information.

### Genetic variation modifying the response to an environmental exposure

Some of the earliest documentation that different people respond to the same environmental exposure in different ways may go back over 2,500 years to the apocryphal story of Pythagoras (570–495 BCE) apparently recognising that only some people developed a fatal reaction to fava beans. The acute haemolytic anaemia causing these deaths is now recognised to be due to the vicine and convicine in the bean inducing reactive oxygen species within cells. While this induction occurs in anyone eating fava beans, in the erythrocytes of individuals with the X-linked glucose-6-phosphate dehydrogenase (G6PD) deficiency, this exposure causes profound haemolysis, anemia, and death.

With the introduction of the 8-aminoquinoline antimalarial drugs in the 20th century, it was noted as early as 1926 that some individuals had fatal responses following their administration [119]. A 1952 study described pamaquine to be associated with haemolytic anaemia in a subset of patients tested: “It may be noted that all six acute hemolytic anemias occurred among 76 pigmented individuals, while only one subacute anemia was observed among 81 white subjects,” with the six “pigmented” individuals separately described: “Five acute hemolytic anemias occurred in negroes and one in a Chinese” [120]. While their terminology is racist, a couple of important lessons emerged from this study, that a subset of people can have severe adverse drug reactions and that this risk can differ in frequency depending on your ancestry (**Box 3**). The susceptibility to haemolytic anaemia following exposure to these antimalarial drugs was subsequently found to be due to G6PD deficiency [121], which is more common in populations originating from regions of the world where malaria is endemic and heterozygosity for the deficiency is protective [122]. In case the repeated mention of malaria causes confusion, the evolutionary selection for G6PD deficiency has nothing to do with the availability of 8-aminoquinoline drugs in the last half century, but instead should have to do with conferring resistance to *Plasmodium falciparum* infection over thousands of years, possibly through the increased phagocytosis of infected erythrocytes that are G6PD deficient [123].

**Box 3. The value of including diverse populations in genetic studies of disease.**

It was emphasised that the example of G6PD deficiency reveals population-specific risks, due to the higher prevalence in populations with a long duration of exposure to malaria. Interestingly, using the Geography of Genetic Variants (GGV) browser [131], we see that the *PNPLA3* I148M pathogenic variant is also very heterogeneous in its frequency in different world populations (**Fig 5**). Because the original Dallas Heart Study cohort was diverse, they recognised the increased frequency of the *PNPLA3* I148M pathogenic variant in the Hispanic subset of their patients [129] who are at higher risk for NAFLD than other ethnic groups. This finding highlights the increasingly appreciated value in studying diverse populations to reveal loci mediating susceptibility to genetic diseases [132]. Increasing the genetic variability of the individuals studied can influence not only how genome-wide association studies (GWAS) perform but also how transcriptomic and epigenomic assays are interpreted. Fortunately, this challenge has been turned into a positive, allowing genetic diversity to inform us about specific loci mediating phenotypes and environmental responses.

The study of how genetic sequence variation between individuals influences the response to drug exposure was founded upon observations like this antimalarial drug association with haemolytic anaemia in G6PD deficiency. The field became known as pharmacogenetics, in which the functions of individual genes could be linked with drug responses, the most straightforward means currently for the delivery of personalised medicine, the tailoring of treatment that takes into account the individuality of the patient.

**Fig 3:**
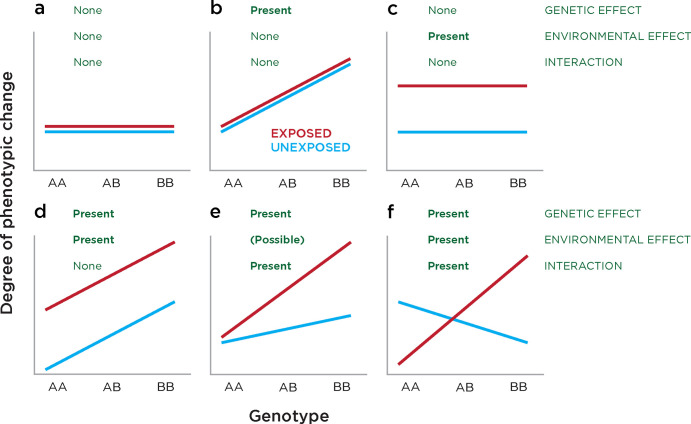
Relating phenotypic change (*y* axis) to genotype (*x* axis, major allele A and minor allele B) in three situations (green), genetic effects, environmental effects, and interactions.

As our ability to explore the DNA sequence polymorphism throughout the genome became facilitated through microarray technologies, studies could be performed that did not need to be anchored by a specific candidate gene but could instead test the entire genome for loci where the response to an environmental exposure was significantly associated with variability at a specific locus. In **Fig 3** (adapted from Dempfle and colleagues [124]), we show examples of the kinds of results that could occur at such a locus. The more common sequence in the population is shown as an A, the less common (minor, alternative) allele as a B. The degree of phenotypic change is plotted on the *y* axis, comparing people with the A allele on both chromosomes (AA), or the minor allele on one (AB) or both (BB) chromosomes. A change in phenotype associated with genotype at this locus should cause the lines to deviate from the horizontal, while an effect of the environment on the phenotype should cause the exposed (red) and unexposed (blue) lines to separate. When the locus itself is helping to mediate the susceptibility to the environmental exposure, you would expect to see both occurring, as well as evidence for interaction (**Fig 3F**). If the phenotypic measure was haemolysis, and we studied a loss of function variant within the *G6PD* gene, and considered only females (who have two copies of this X chromosome gene), we should find the blue line to slope gently upwards, as there is a chronic, low-level hemolysis in individuals with G6PD deficiency. Following exposure to fava beans or an 8-aminoquinoline antimalarial drug, the red line should separate strongly upwards in the AB and BB individuals, demonstrating the interaction at this locus. Due to the interaction of genotype and phenotype via environmental interactions, studying them together could be a more powerful method to study the contribution of DNA methylation to disease outcomes. Indeed, DNA methylation at variably methylated regions in neonatal cord blood could be explained by both genetic and environmental effects studied in an integrated model [125].

**Fig 4:**
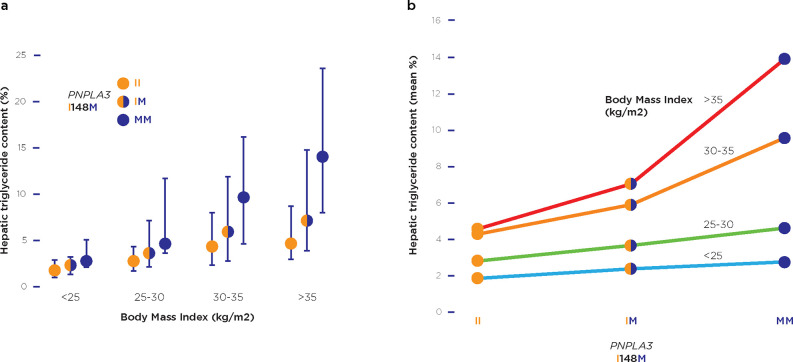
(**a**) The interaction of genotype and body mass index to cause MAFLD (quantified by hepatic triglyceride content, *y* axis) is vividly revealed using data from the Dallas Heart Study [116]. (**b**) Replotting the same data using the format of **Fig 2** to show the combined genetic and environmental influences in MAFLD. The WebPlotDigitizer tool (https://automeris.io/WebPlotDigitizer/) was used to extract the raw data from the source image in the published work [115], in the disease.

Gene-environment-wide interaction studies (GEWIS) typically emerged from GWAS, which by themselves attempt to link phenotype only with genotype (as would be exemplified in **Fig 3B**). When the environmental exposure information is available for the people studied, the extra dimension of GEWIS can be added and allows exposures beyond medications to be studied. The exposure does not need to be pharmacological. The field of gene–environment (GxE) interaction research involves major methodological and statistical challenges [126], including the ability to detect these interactions with confidence [127] and suffers from imprecision of the use of terms describing the field [124], but has been successful in many associations, in particular when involving genes with metabolic functions [128,129]. While subsequent GWAS have identified further risk loci [47], a further analysis of the Dallas Heart Study participants revealed the striking results shown in **Fig 4A** (reproduced from [130]). This figure was used to demonstrate vividly how the *PNPLA3* genotype interacts with BMI. **Fig 4B** illustrates how BMI, when taken as a proxy for its causative environmental exposures, generates a plot comparable with the models in **Fig 3**, as a way of illustrating the effects of both genetic and environmental factors

**Fig 5:**
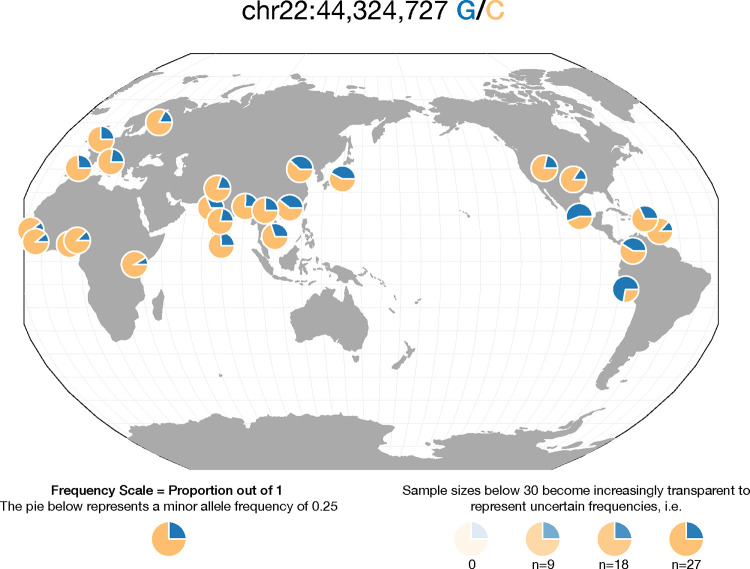
The distribution of the *PNPLA3* I148M variant (blue) worldwide shows the greatest enrichment in populations from Central and South America, while being a common variant in all populations.

### How to test for influences by DNA sequence variants

An issue with any GWAS, or its extension to study environmental effects through GEWIS and GxE approaches, is that it does not point us to a specific locus at nucleotide resolution when it finds a phenotypic association. Instead, it implicates a haplotype, usually at least tens of kilobases in size and containing potentially thousands of variants [133]. To refine the search for the causative locus within the haplotype, one strategy has been to exploit the differences in phenotypic susceptibility between individuals of different ancestries to fine-map the region [134]. Another strategy uses the effects described earlier for DNA sequence variability influencing transcription and its regulatory mechanisms, identifying the subset of functional variants in these regions, allowing them to be prioritised as potentially mediating the phenotype.

The most studied effect of functional variants is on gene expression. By treating the level of expression of a gene as a quantitative phenotype and correlating it with genetic variation, a locus where variation of the DNA sequence is associated with a change in gene expression can be defined. The sequences tested can be on the same chromosome and relatively close (typically <1 Mb) to the gene whose expression level is measured, a *cis* relationship, or further away or on another chromosome, a *trans* relationship. The outcome sought is a change similar to that in **Fig 2**, with the variant described as an eQTL and the target gene an eGene. By identifying the eQTLs in a haplotype implicated by an EWAS, the number of variants can be reduced from hundreds to a very limited number [133]. An even more powerful way of refining the search for causal variants is through studies that look for effects of a variant present on one but not the other allele, leading to an imbalance of expression of a linked gene, referred to as “allele-specific expression” [135]. This helps to safeguard against attributing function to a variant in a region of the genome in high linkage disequilibrium with neighbouring variants, one of which may instead be mediating the functional effect.

Something interesting happens when you look for eQTLs after challenging the cell with an exposure. The groundbreaking study that revealed “response” eQTLs used an exposure of dendritic cells to infection by *Mycobacterium tuberculosis*. The authors found that while most eQTLs remained the same before and after exposure, a subset was present only in the uninfected or in the infected cells, and were described as response eQTLs. Within the panel of eQTLs genome wide, the response eQTLs were enriched at loci identified by GWAS as being involved in susceptibility to tuberculosis [136].

Response eQTLs have now been identified for multiple different exposures and cell types. Human monocytes have been tested following Toll-like receptor 4 (TLR4) stimulation [137], human dendritic cells were exposed to *E*. *coli* lipopolysaccharide, influenza, or interferon-β (IFN-β) [138], human monocytes were treated with interferon-γ (IFN-γ) or lipopolysaccharide [139], monocyte-derived macrophages were infected with *Listeria monocytogenes* or *Salmonella typhimurium* [140], while primary monocytes were exposed to ligands activating Toll-like receptor pathways (TLR1/2, TLR4, and TLR7/8) and to influenza virus, in samples from Africans and Europeans [141]. A study that exposed human macrophages to IFNγ, *Salmonella enterica* serovar Typhimurium, or a combination of the two exposures implicated certain TFs in mediating the response to infection, with a primary effect on PU.1 and secondary effects on stimulus-specific TFs, such as NF-κB and STAT2 [142]. A study of whole blood from 1,000 individuals, using exposures to three bacteria, a fungus, a live virus, and a superantigen, demonstrated that variability in responses between individuals was less influenced by age and sex and more by genetic factors, identifying response eQTLs enriched at loci implicated in autoimmune and inflammatory disorders [143]. Allele-specific expression has also proven valuable for discovering response eQTLs to factors such as BMI and exercise in large observational cohorts [144].

As well as these studies of infection and the immune system, we performed a study identifying response eQTLs following exposure of cardiomyocytes to anthracycline [2]. These response eQTLs were more enriched than preexposure eQTLs for loci implicated by GWAS for anthracycline-induced cardiotoxicity. The approach to study allele-specific expression has also been successfully scaled to a high number (50) of environmental exposures in five different cell types, revealing a large number of genes with GxE effects [145]. What all of these studies have in common is that they represent in vitro exposures by agents known to induce responses by the cells used. An obvious question that arises is how to apply these approaches to other diseases. Maintaining our focus on NAFLD, earlier, we described the environmental exposures to the individual may end up translating into exposures to the cells of the liver, including the effects of lipotoxicity on mixed cells types within the liver. We need to ask whether we can study one cell type in isolation, or whether effects of an exposure require the physical relationship between the cells that compose the normal liver. Rather than sampling primary liver cells from human subjects, which has substantially more risk than a blood draw to sample immune cells, we can use the approach of the anthracycline/cardiomyocytes study, which generated cardiomyocytes from induced pluripotent stem cells (iPSCs) from multiple individuals [2]. iPSCs can also be used to generate organoids that contain many of the cell subtypes of an organ, including liver organoids [146], which may be another avenue worth pursuing in studies of responses to exposures, although we highlight the caution that the field of organoid research is still in its early stages [147].

Once we have our exposure and cell system in place, we can move to molecular studies. The genotyping of samples is a foundation for understanding how genetic variability influences environmental responses and the transcriptional and epigenomic data generated. Furthermore, as mentioned above, the genotypic contribution to disease outcome is likely expressed in a tissue- and cell-specific manner [93]. Genotype information can be inferred from DNA methylation microarrays [148], while it has also been found that Gap Hunter can reveal ancestry information [86]. However, these approaches generates much less genotyping information than more mainstream approaches such as the use of microarrays that represent common variants in the genome, ideally designed to be as informative as possible across diverse populations [149]. An alternative is low-coverage whole genome sequencing, at a depth that is insufficient to identify every variant in the genome but allows the identification of many common variants through imputation methods that leverage large reference panels, and the additional revelation of some lower frequency variants that would not be detected by microarrays [150].

The next molecular assay is typically gene expression analysis, allowing eQTL identification, as described already. However, there are other molecular assays that reveal the effects of functional variants. While we have described meQTLs earlier as loci responding to functional variants by changing their DNA methylation, in practice, these have not been used to test for environmentally responsive loci in a way comparable with eQTL studies. Chromatin accessibility QTLs (caQTLs) represent an intriguing alternative that is understudied at present. An ingenious pooling approach was used to reveal caQTLs in lymphoblastoid cell lines from 1,000 individuals from 10 populations, revealing population-specific caQTLs [151], although this study did not involve any in vitro exposure. A recent, groundbreaking study combined the comparison of cells before and after exposure, testing multiple immune cell types, and identifying both response eQTLs and response caQTLs. The authors found evidence for function of candidate causal variants that would have been undetectable using more mainstream approaches studying resting cells [152]. A problem with eQTLs is that the expression of a gene is generally influenced by multiple *cis* regulators [153], making it difficult to link local sequence variation with the quantitative trait of gene expression. Chromatin accessibility, on the other hand, is likely to vary at the locus containing the functional variant, lending itself to allele-specific studies [151,154]. A shift in focus from eQTLs to caQTLs may be fruitful for functional variant analyses.

Finally, it should be borne in mind that the relatively common variants that we can identify through microarray or low-coverage whole genome sequencing with imputation may only contribute a small proportion of variability of gene expression. Even with detailed haplotype information (in mostly European populations), the imputation can only predict variants down to a frequency of 1/1,000 (10^−3^) [155]. A study of 360 LCLs derived from European individuals combined deep whole genome sequencing and RNA sequencing to identify which variants influenced the heritability of levels of gene expression. Approximately 90% of the heritability was found to be associated with sequence variants that occurred only once in the cohort (singletons) and at a minor allele frequency of <0.01% in the gnomAD database [156]. If we assume that the variants identified in this study represent those with effects on DNA methylation (meQTLs) and chromatin accessibility (caQTLs), we can expect that only a small proportion of variants causing changes in expression, DNA methylation and chromatin accessibility will be revealed by current approaches, that deep whole genome sequencing will reveal many more variants affecting these molecular phenotypes, and that most functional variants will be present in the genome in a heterozygous state because of their population rarity.

### Insights from the epigenome into cell signalling

To close the circle, we return to the use of epigenomic assays but now dissociated from their ability to reveal information about DNA sequence variants in mediating differences between individuals in their responses to environmental influences. A question that is often left unaddressed in epigenomic studies is why specific loci undergo changes, whether of DNA methylation or chromatin accessibility. We have made the point previously that such targeting implies a primary role for TFs [65]. The reason that functional variants have their properties appears often to be due to the effect of the DNA sequence to change local TF binding [154]. In a recent study of oestradiol’s effects on the ventral hippocampus of female mice, we showed that the hormone acts on a cell surface receptor to initiate a cytoplasmic cell signalling cascade that culminates in the activity of the Egr1 TF to change chromatin structure and gene expression [157]. The Alasoo and colleagues’ study described earlier implicated specific TFs in mediating the response eQTLs they identified [142]. Of the environmental influences with potential effects on epigenetic regulators listed in **Table 1**, hypoxia [158], hyperglycaemia [159], ethanol [160], and lactic acid [161] are good examples of environmental cellular stresses known to influence cell signalling pathways, with potential consequences on TF activity and nuclear localisation. With this TF-centred perspective, we can return to some of the foundational studies in the field of environmental epigenomics and ask whether they could be viewed alternatively with a TF-centric perspective.

One very understudied area of potential importance is the PTM of TFs. It is known that some TFs can be acetylated or deacetylated by the same enzymes that act on histones [162], with methylation and demethylation likewise mediated by histone-modifying enzymes [163]. The effects of environmental exposures that disrupt histone acetylation, methylation, or other PTMs could also therefore be acting on TFs. The paradigm of dietary folic acid having effects on transcriptional regulation may not be solely due to its effects to augment DNA methylation but could be mediated through the property of the folic acid receptor to act as a TF [164]. The phenomenon of temperature-dependent sex determination took over 50 years to find that the transcription factor *Dmrt1* (*doublesex and mab3-related transcription factor 1*) is likely to be a primary mediator of the temperature response in another amphibian, the red-eared slider turtle *Trachemys scripta* [165]. Considering the effects of the environment as being mediated through TFs helps to explain the sequence specificity of environmental responses by the epigenome.

If TFs are central in directing the response by the cell to an environmental exposure, the next question is what controls the TFs? Some are directly bound in the nucleus by ligands, the nuclear receptors [166], but many TFs act in response to cell signalling pathways [167]. This suggests that transcriptomic and epigenomic assays are defining two sets of information following an environmental exposure. Typically, we look for a coherence in the loci where changes are occurring in the genome, whether genes changing expression, or the genes linked to loci where DNA methylation or chromatin states are changing. The coherence is expressed in terms of the gene properties (through overrepresentation of specific gene ontology terms) or through known interactions between the protein products of genes.

**Fig 6:**
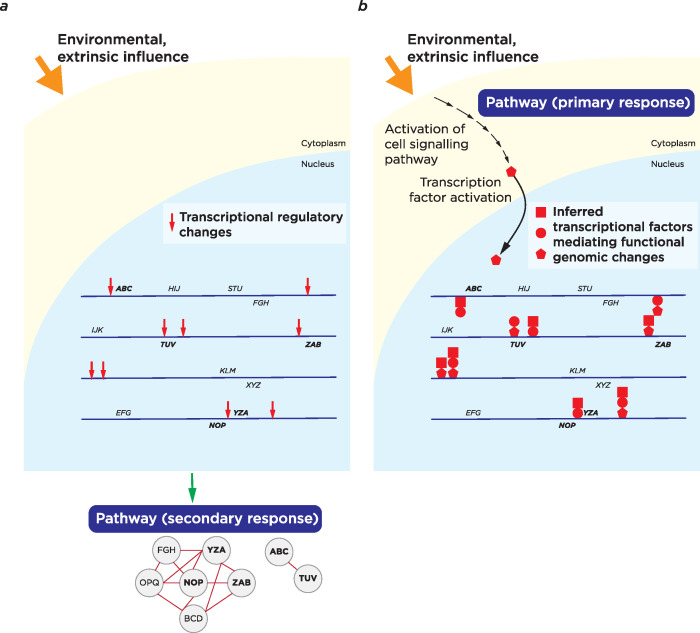
(**a**) When testing the response to an extrinsic influence using genomic approaches, we typically identify the loci at which changes are most pronounced and attempt to understand how they have biological coherence by studying the genes implicated for their biological properties, including protein–protein interactions. While this is part of the cellular response to the extrinsic perturbation, an alternative perspective is shown in (**b**), considering the role of TFs to select loci for altered function and, in turn, the cell signalling pathways that regulate the TFs. The activation of TFs in this model would be the primary response to the extrinsic influence, with the transcriptional regulatory changes a secondary event.

If, however, this represents downstream of TF activity selecting these loci in the genome, it could be said that this represents a secondary response to the environmental stimulus. By identifying the TF(s) mediating the response and working upstream, we can define the primary response to the environmental influence. This logic was used to develop SPAGI (Signalling Pathway Analysis for putative Gene regulatory network Identification), a tool that infers from TFs implicated in a transcriptional response the pathways that activated those TFs in the first place [168]. We illustrate these ideas of secondary and primary pathway responses in **Fig 6**, representing a further way of exploiting epigenomic and transcriptomic information when studying environmental responses by the cell. With a goal of defining targets for therapeutic intervention, defining cell signalling pathways involved in environmental responses offers clear opportunities, but it should be noted that the TFs themselves are no longer considered “undruggable” [169–171] and that, in the case of NAFLD and NASH, there are some nuclear receptor TFs that have been targeted for therapy (Peroxisome proliferator-activated receptor (PPAR) proteins and the Farnesoid X receptor (FXR) [172]). Epigenomic assays, used with the idea that they reveal cell signalling and TFs mediating environmental responses, could be valuable in defining targets for therapeutic intervention.

With a TF-centric perspective that leads us to consider the role of cell signalling, we can return to a fundamental question about epigenetics and heritability—does the cell signalling state of the parent cell influence that of daughter cells? This would represent a further mechanism for inheriting cellular properties through cell division. There is evidence for such a mechanism. Alterations of parental cell stress or mitogenic activity in parent cells has been found to influence cell cycle commitment in the daughter cells, mediated by transmission of the p53 protein and the cyclin D1 (*CCND1*) mRNA through mitosis [173]. It is probably reasonable to consider this as an example of a broader phenomenon of nongenetic heritability through cell division, although probably an uncomfortable fit for those who would describe epigenetic heritability purely in terms of nuclear information.

## Conclusions and future directions

The study of the epigenome and its response to environmental exposures can be much more encompassing than current models would indicate, making these studies more complex but also more promising and exciting in terms of the potential insights to be gained. As a field, our initial hope was that focusing studies on the regulators of the genome would be enough to give direct clues to the mechanisms and outcomes of environmental exposures. While this remains possible, in many cases, these studies are more likely to be revealing the effects of variation of DNA sequence, or of cell subtype proportions, and the effects of TFs. The mainstream interpretation of EWAS that defines these influences as sources of error is excessively restrictive. By embracing these “spurious” influences instead as sources of insights into the physiological or pathological effects of environmental exposures, we expand the opportunity to discover more mechanisms underlying the associated phenotype. Epigenomics assays can become a greater part of the repertoire of approaches being used to follow up GWAS to identify the genetic loci involved. To gain new insights into human cell and tissue types that are normally inaccessible, we can leverage advances in iPSC differentiation to create many different cell types or even organoids, permitting the detailed dissection of molecular events responding to in vitro exposures in these highly controlled systems. Additionally, we have the opportunity to use epigenomic approaches to not only reveal the TFs central to mediating environmental responses, but also to infer their upstream cell signalling regulators, revealing possible targets for therapeutic interventions.

This more expansive approach to the use of epigenomics to understand environmental influences in disease comes with clear challenges. The issue of very rare genetic variants influencing environmental responses, and the resulting phenotypes of the individual, their cells and the molecular regulators is a significant problem. The identification of the ultrarare variants that may mediate a significant proportion of these interindividual differences requires deep whole genome sequencing. However, this creates another opportunity for epigenomic approaches—while the individual DNA sequence variants will be rarely observed more than once even in large cohorts, many different rare variants at a locus can have the convergent outcome of a change in a functional property of the locus, whether chromatin accessibility, DNA methylation, or an effect on nearby gene expression. Multiple different rare variants at a locus are likely to converge functionally as the same kind of change in a functional genomic property. The most productive strategy is therefore to associate the polymorphism of molecular genomic phenotypes with exposures or cellular/organismal phenotypes as the primary association, which should be relatively more easily detected. The more typical approach that links DNA sequence variability with the molecular genomic phenotype thus becomes the secondary association. This convergence of multiple rare variant effects on a molecular genomic outcome in effect “collapses” the multiple DNA sequence variants into a common outcome to increase association power.

There are deficiencies in our insights into the repertoire of DNA motifs bound by TFs, the upstream regulatory influences upon TFs by cell signalling pathways, and the related issue of the types, mediators, and effects of PTMs of TFs. Any in vitro studies will need to be designed in a way that represents the best guess about the exposures at the cellular level in vivo and the one or more cell types responding to mediate the disease or other phenotype. Animal models will continue to have major value as a parallel to these direct studies of human cells. Broadening the repertoire of pluripotent stem cell resources to represent more diverse racial and ethnic groups with distinctive risks of environmentally responsive phenotypes will be another pressing need.

Finally, it should be borne in mind that just because GWAS or the extended GEWIS and GxE studies do not explain all susceptibility to a disease, this is not by itself a justification for performing epigenomic studies of disease. The gap between what GWAS findings can explain and the heritability estimated from twin and other studies has been described as “missing heritability.” To fill this gap, environmental interactions are invoked, and epigenetic dysregulation as a consequence of the environmental influences or as a separate source of variability is also considered. Missing heritability may be due to many factors, including the inflation of the estimate of heritability from twin studies, limited sample size, the polygenicity of phenotypes [174], and the rarity of the genetic variants causing the conditions [175]. What we tend to overlook is that chance is likely to be an additional factor in phenotypes [176], but that should be easily embraced by those interested in epigenetics, as the ball rolling down Waddington’s epigenetic landscape was not predestined to end up in a specific creode following a series of bifurcations; the future lineage commitment of the cell being represented was probabilistic and, therefore, subject to random variability. Nondeterminism is therefore at the core of the original idea of an epigenetic model for phenotypic variability.
